# Serum ionized magnesium acts as an independent protective factor against bone erosion in patients with gouty arthritis: a cross-sectional study

**DOI:** 10.3389/fendo.2024.1375871

**Published:** 2024-09-30

**Authors:** Yixuan Li, Yahao Wang, Lili Xu, Chuanfeng Liu, Jiufa Cui, Yajing Huang, Shufa Li, Yangang Wang, Bingzi Dong

**Affiliations:** ^1^ Department of Endocrinology, The Affiliated Hospital of Qingdao University, Qingdao, China; ^2^ Department of Endocrinology, Huashan Hospital, Fudan University, Shanghai, China; ^3^ Department of Hematology, The Affiliated Hospital of Qingdao University, Qingdao, China; ^4^ Department of Radiology, The Affiliated Hospital of Qingdao University, Qingdao, China

**Keywords:** bone erosion, magnesium, gouty arthritis, gout, biochemical markers of bone turnover

## Abstract

**Background:**

Gouty arthritis is a common inflammatory arthritis. The recurrent gout attacks severely damage the joint’s function, lead to bone erosion, and affect bone metabolism. The role of magnesium (Mg) ions in bone homeostasis has been recognized, whereas its specific relationship with gouty bone erosion remains unclear. This study examined the association between serum ionized Mg levels and bone erosion in patients with gout arthritis.

**Methods:**

A total of 769 patients with gout arthritis were included in the study. Participants were classified into four groups based on the quartiles of the serum ionized Mg level. Logistic regression analysis assessed the association between serum ionized Mg and bone erosion.

**Results:**

Compared to patients without bone erosion, serum ionized Mg levels were lower in gout patients with bone erosion (p<0.001). When dividing serum ionized Mg into quartiles, the prevalence rate of bone erosion in group Q1, representing the patients with the lowest serum ionized Mg levels, was notably higher than in Q2, Q3, and Q4 (60.2% vs. 43.6%, 45.6%, 40.3%, p<0.001). Multiple logistic regression analysis revealed that patients in Q2-Q4 had a lower odds ratio (OR) of bone erosion compared to those in Q1 (ORs were 0.520, 0.533, and 0.411 in Q2-Q4, respectively, p<0.001).

**Conclusion:**

The incidence of bone erosion is higher in gout arthritis patients with lower serum ionized Mg levels. High serum ionized Mg levels may be an independent protective factor for bone erosion in gout arthritis. Thus, Mg supplementation may be a promising approach to prevent or slow down the development of bone erosion in gouty arthritis.

## 1 Introduction

Gouty arthritis is a common inflammatory arthritis caused by the deposition of monosodium urate (MSU) crystals in and around the joints (1). Over the past two decades, there has been a consistent global increase in the prevalence and incidence of gout (2). Clinically, patients with gout often manifest acute joint inflammation (3). Frequent episodes of gout can significantly impair joint function and may result in bone erosion and cartilage deterioration, thereby impacting overall bone health (3, 4). Gout patients may be at an increased risk of bone resorption and fractures (5, 6). Therefore, gout can significantly affect bone tissue, a topic that merits additional investigation, especially in the context of current concerns about bone health.

Bone erosion refers to the localized destructions of bone (7). It typically starts with the cortical bone and involves the destruction of the natural barrier between the extraskeletal tissue and the intertrabecular spaces of the bone marrow cavity (7). Bone erosion is one of the characteristic radiographic hallmarks of chronic gouty arthritis (8). Tophus erosion plays a pivotal role in the development of bone erosions in gouty arthritis (8). In the context of gout disease, bone erosion is irreversible once it occurs (9). The early detection and assessment of bone erosion are conducive to decreasing the risk of joint function disability (10). At present, assessment of bone erosion is mainly performed by the modified Sharp/van der Heijde (SvdH) erosion scoring system based on Dual-energy computed tomography (DECT) images (11, 12). Some studies found that peculiar imaging findings, such as urate deposition (UD), MSU deposition, and tophus deposition, are strongly associated with bone erosion (12–14). However, few studies investigated the relationship between biochemical parameters and bone erosion, in particular for the ions closely related to bone metabolism.

Mg^2+^, as a divalent metal ion, is the second largest intracellular cation after potassium (15). Approximately 60% of the Mg in the human body is stored in the skeleton (16). Available research has demonstrated that Mg plays an important role in maintaining bone health (17). Deficiency of Mg may lead to osteoporosis and increased risk of fractures (17–19). Furthermore, Mg has also been confirmed to inhibit osteoclastogenesis and promote osteoblast differentiation, proliferation, migration, and attachment, to support bone minerlization (20, 21). A recent large retrospective study of 14,566 American adults noted that dietary Mg intake was inversely correlated with osteoporosis, and inadequate Mg intake provides a basis and reference for the early monitoring and interventions of osteoporosis (22). It has been well acknowledged that Mg homeostasis is essential for maintaining the balance of bone formation and bone resorption (23). Although a few studies describe the relationship between Mg homeostasis and bone health, few tend to focus on joint damage in patients with gout. Given the importance of bone erosion in predicting bone abnormalities in gout, it is of interest to explore whether bone erosion is also affected by Mg levels.

In this study, we conducted a cross-sectional study to examine the association between serum ionized Mg levels and bone erosion in patients with gout arthritis.

## 2 Methods

### 2.1 Study population

**Figure 1:**
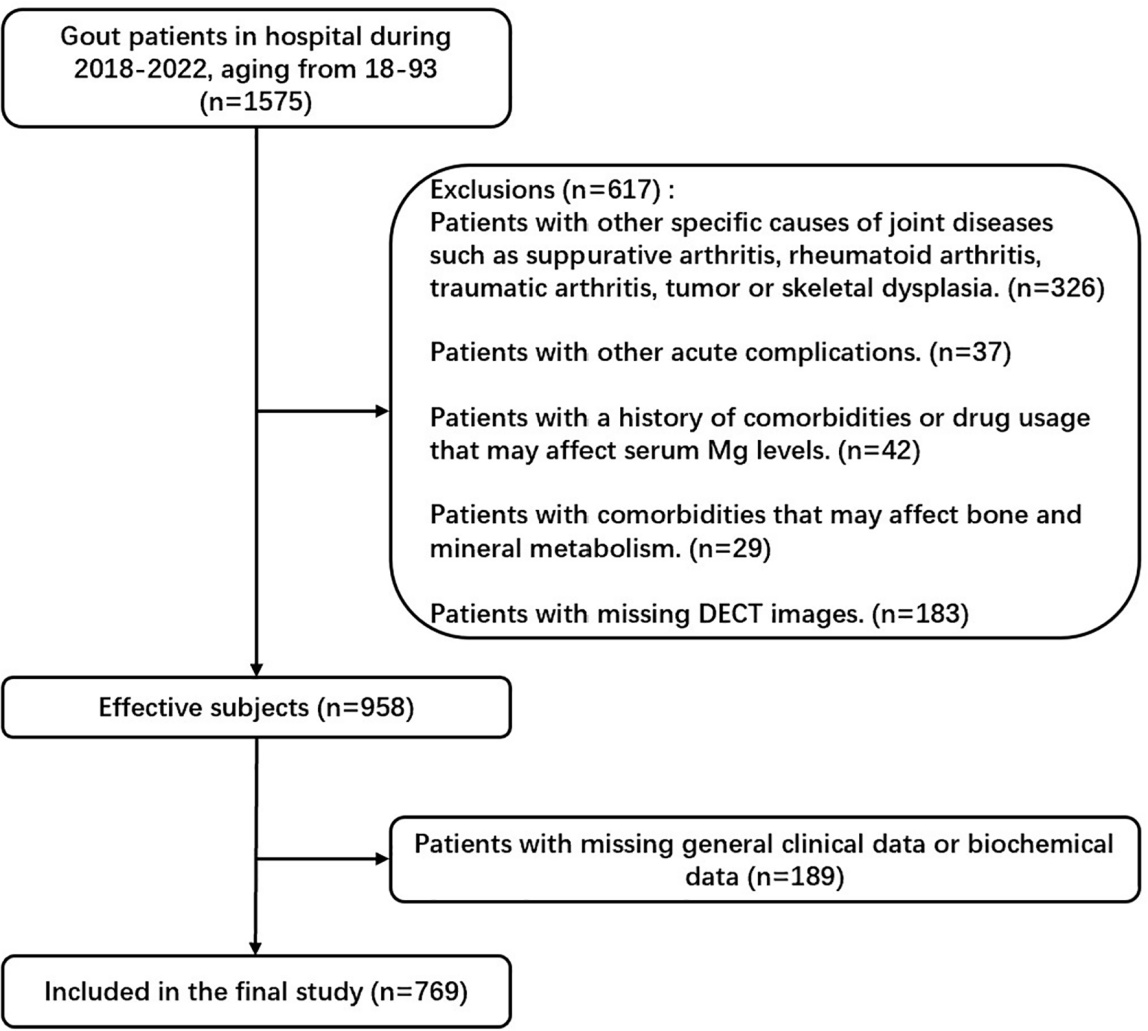
Flowchart of study participants. In this study, we excluded patients with specific joint diseases, acute complications, comorbidities or medications affecting serum Mg levels, bone or mineral metabolism disorders, and incomplete clinical data.

This study enrolled 1575 gout patients, who attended the Affiliated Hospital of Qingdao University (Qingdao, China) between January 2018 and September 2022. A total of 769 patients with gouty arthritis who met the inclusion criteria and did not meet the exclusion criteria were enrolled (**Figure 1**). The inclusion criteria were: 1) All the patients met the 2015 ACR/EULAR classification criteria for gout^11^. 2) All the patients underwent DECT scans of joints including interphalangeal joints, wrists, elbows, ankles, and knees. The exclusion criteria were: 1) Patients with other specific causes of joint diseases such as suppurative arthritis, rheumatoid arthritis, traumatic arthritis, tumors, or skeletal dysplasia. 2) Patients with other acute complications. 3) Patients with a history of comorbidities or drug usage that may affect serum Mg levels include those taking diuretics, laxatives, or oral/intravenous magnesium supplements, those with alcohol abuse, users of proton pump inhibitors, those receiving immunosuppressant or chemotherapy agents such as Cisplatin, and so forth. Patients with comorbidities that may affect bone and mineral metabolism, including hyper-/hypoparathyroidism, hypo-/hypercalcemia, salt-wasting renal tubule disorders, metabolic bone disorders, hypoalbuminemia, severe insulin resistance, malnutrition and cachexia, diarrhea, renal dysfunction, and a history of gastrointestinal operations, were excluded from the study. 4) Patients with incomplete clinical data. The ethics committee of the Affiliated Hospital of Medicine College Qingdao University approved the study. All participants provided written informed consent.

### 2.2 Data collection

Anthropometric parameters included age, sex, gout duration, height, weight, waist, hip, and blood pressure. Body mass index (BMI) was calculated as weight (kg)/height squared (m^2^). Blood samples were drawn in the morning after an overnight fast (>10 h). Blood test indicators contained glycated hemoglobin (HbA1c), fasting plasma glucose (FPG), C peptide, fasting insulin, free fatty acid (FFA), serum uric acid (SUA), serum creatinine (SCr), serum ionized calcium (Ca), serum ionized Mg, serum phosphorus (P), alkaline phosphatase (ALP), 25-hydroxyvitamin D3 (25OHVD3); procollagen type I N-terminal propeptide (PINP); β-C-terminal telopeptide of type 1 collagen (β-CTX); osteocalcin (OCN). HbA1c was assessed by high-performance liquid chromatography (MQ-2000PT, China). Serum FPG, FFA, ALP, SUA, and SCr were measured by the Hitachi 7600 automatic biochemical analyzer. The serum levels of Ca, Mg, P, 25OHVD3, PINP, β-CTX, and OCN were detected by a Roche E801 analyzer. Bone mineral density (BMD) was assessed by dual energy X-ray absorptiometry (DXA) (Norland, USA). Osteoporosis is diagnosed as the T score is =<−2.5, or/and with the presence of fragility fracture, according to the guidelines of diagnosis and treatment of osteoporosis (24).

### 2.3 Assessment of bone erosion

**Figure 2:**
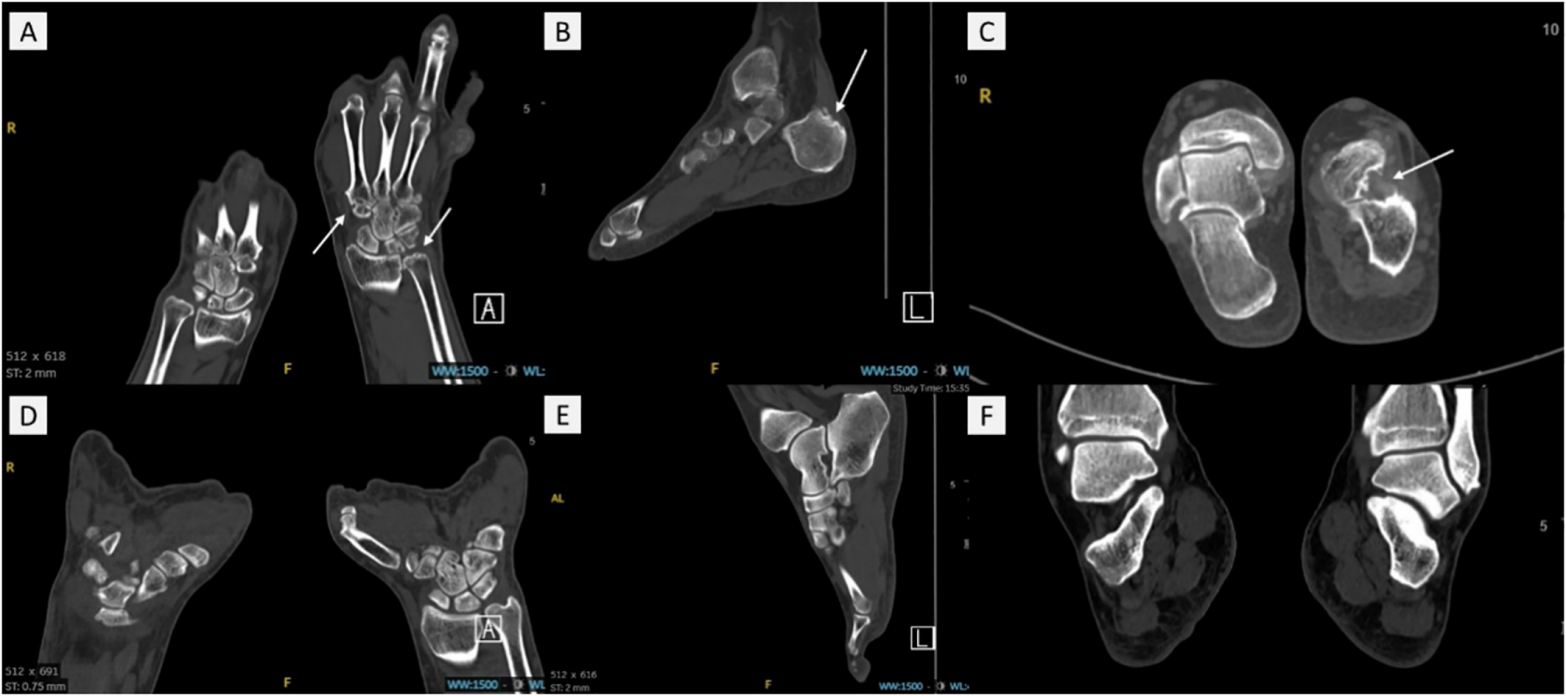
The DECT images of gout patients with bone erosion and without bone erosion. **(A)** and **(D)** showed the hand/wrist area. **(B, C, E)** and **(F)** showed the foot/ankle area. White arrows present bone erosion in **(A–C)** No bone erosion was observed in **(D–F)**. .

Bone erosion was assessed using DECT, defined as cortical destruction with bone contour defects in at least two perpendicular planes (25). (**Figure 2**). The DECT images of all participants were evaluated independently by two musculoskeletal radiologists who were fully blinded to the blood test results. The patients were grouped into two groups: those without bone erosion and those with bone erosion.

### 2.4 Statistical analysis

Statistical analysis was performed with SPSS 26.0 (SPSS Inc., Chicago, IL, USA). Figures were plotted by GraphPad 8.0.2. All continuous variables were firstly evaluated for normality using the Kolmogorov–Smirnov test. Continuous variables were presented as mean ± standard deviation (SD) for normally distributed data or median with interquartile range (IQR) for non-normal distributed data. Categorical variables were displayed as percentages (%). Differences between the group without bone erosion and the group with bone erosion were tested using Student’s t-test for normally distributed continuous variables, the Mann–Whitney U test for non-normal continuous variables, or the Chi-square test for categorical variables. Group by quartile of serum ionized Mg: Q1: serum ionized Mg ≤0.83 mmol/L; Q2: 0.83<serum ionized Mg <0.88 mmol/L; Q3: 0.88≤serum ionized Mg <0.92 mmol/L; Q4: serum ionized Mg ≥0.92 mmol/L. Continuous variables with normal distribution were compared using a one-way analysis of variance (ANOVA) test. For non-normally distributed variables, the Kruskal-Wallis test was used. The chi-square test was still used to compare categorical variables between multiple groups. The correlations between clinical characteristics and serum ionized Mg level were analyzed by Spearman correlation. Multiple logistic regression analysis was adopted to determine the association between serum ionized Mg levels and bone erosion risk. Significance was indicated with *p<0.05.

## 3 Results

### 3.1 Clinical characteristics of gouty arthritis patients with or without bone erosion

A total of 769 patients were enrolled in this study. Among them, 95.4% were male, and 4.6% were female. Age ranged from 18 to 93 years. The duration of gout varied from 3 months to 45 years. The clinical characteristics of gout participants are presented in **Table 1**, including 403 (52.4%) patients without bone erosion and 366 (47.59%) patients with bone erosion.

**Table 1:** Clinical characteristics of gout participants without bone erosion and with bone erosion.

Characteristics	Without bone erosion N=403	Bone erosion N=366	P-value	Reference range
General clinical data				
Age, y	48.9 ± 15.8	53.8 ± 15.1	< 0.001*	–
Gender, male, %	386(95.8)	348(95.1)	0.642	–
Gout duration, y	6(2,12)	9(5,15)	< 0.001*	–
Height, cm	174.7 ± 6.7	173.2 ± 6.5	0.002*	–
Weight, kg	87.4 ± 16.0	84.7 ± 17.7	0.024*	–
BMI, kg/m^2^	28.6 ± 4.3	28.1 ± 4.8	0.133	–
Waist, cm	101.3 ± 11.6	101.3 ± 13.7	0.955	–
Hip, cm	104.5 ± 8.4	104.5 ± 11.3	0.549	–
SBP, mmHg	138 ± 16	140 ± 18	0.065	–
DBP, mmHg	85 ± 12	86 ± 13	0.310	–
Radiographic features				–
Number of involved joints	1.7 ± 2.0	3.3 ± 3.1	< 0.001*	–
Joint effusion, %	288(56.6)	206(56.3)	0.935	–
Synovial thickening, %	205(75.6)	216(80.9)	0.140	–
Tophi in soft tissues, %	56(13.9)	67(18.3)	0.096	–
Biochemical profile				
HbA1c, %	5.7(5.4,6.2)	5.8(5.5,6.3)	0.889	3.6-6.0
FPG, mmol/L	5.00(4.51,5.78)	5.12(4.53,5.84)	0.826	3.90-6.16
C peptide, ng/mL	3.62 ± 1.57	3.81 ± 2.03	0.154	1.1-4.4
Fasting insulin, uIU/mL	13.95(9.56,18.77)	13.32(8.48,17.62)	0.146	2.6-24.9
FFA, mmol/L	0.41 ± 0.16	0.41 ± 0.17	0.578	0.1-0.60
SUA, umol/L	453 ± 119	466 ± 119	0.124	89.2-416
SCr, umol/L	74(62,87)	75(63,93)	0.068	31-132
Ca, mmol/L	2.14 ± 0.18	2.18 ± 0.18	0.003*	2.11-2.52
Mg, mmol/L	0.89 ± 0.07	0.86 ± 0.08	< 0.001*	0.75-1.02
P, mmol/L	1.21 ± 0.19	1.22 ± 0.18	0.658	0.85-1.51
Bone metabolism markers				
ALP, U/L	64.0(54.0,74.0)	68.0(57.0,89.8)	0.029*	45-125
25OHD3, ng/mL	16.1(11.5,22.1)	15.8(10.9,20.7)	0.412	Summer 15.7-60.3 Winter 8.8-46.3
PINP, ng/mL	37.3(26.9,52.6)	46.6(33.2,65.8)	0.002*	9.06-76.24
β-CTX, ng/mL	0.51(0.28,0.75)	0.56(0.39,0.87)	0.025*	0.043-0.783
OCN, ng/mL	12.24(9.2,18.0)	15.35(11.1,20.5)	0.012*	6-24.66
Osteoporosis, n (%)	69(17.1)	67(18.3)	0.667	–

A total of 239 patients with bone metabolic markers results were analyzed, including 119 patients without bone erosion and 120 patients with bone erosion.

Independent sample t-test, Mann–Whitney U test, or Chi-square test.

*p<0.05 indicates a statistical difference.

Normally distributed variables are expressed as mean ± standard deviation. Non-normal variables are expressed as median (IQR). Categorical variables are expressed as percentages (%).

BMI, body mass index; SBP, systolic blood pressure; DBP, diastolic blood pressure; HbA1c, glycated hemoglobin; FPG, fasting plasma glucose; FFA, free fatty acid; SUA, serum uric acid; SCr, serum creatinine; Ca, serum ionized calcium; Mg, serum ionized magnesium; P, serum phosphorus; ALP, alkaline phosphatase; 25OHVD3, 25-hydroxyvitamin D3; PINP, procollagen type I N-terminal propeptide; β-CTX, β-C-terminal telopeptide of type 1 collagen; OCN, osteocalcin; IQR, inter-quartile range.

**Figure 3:**
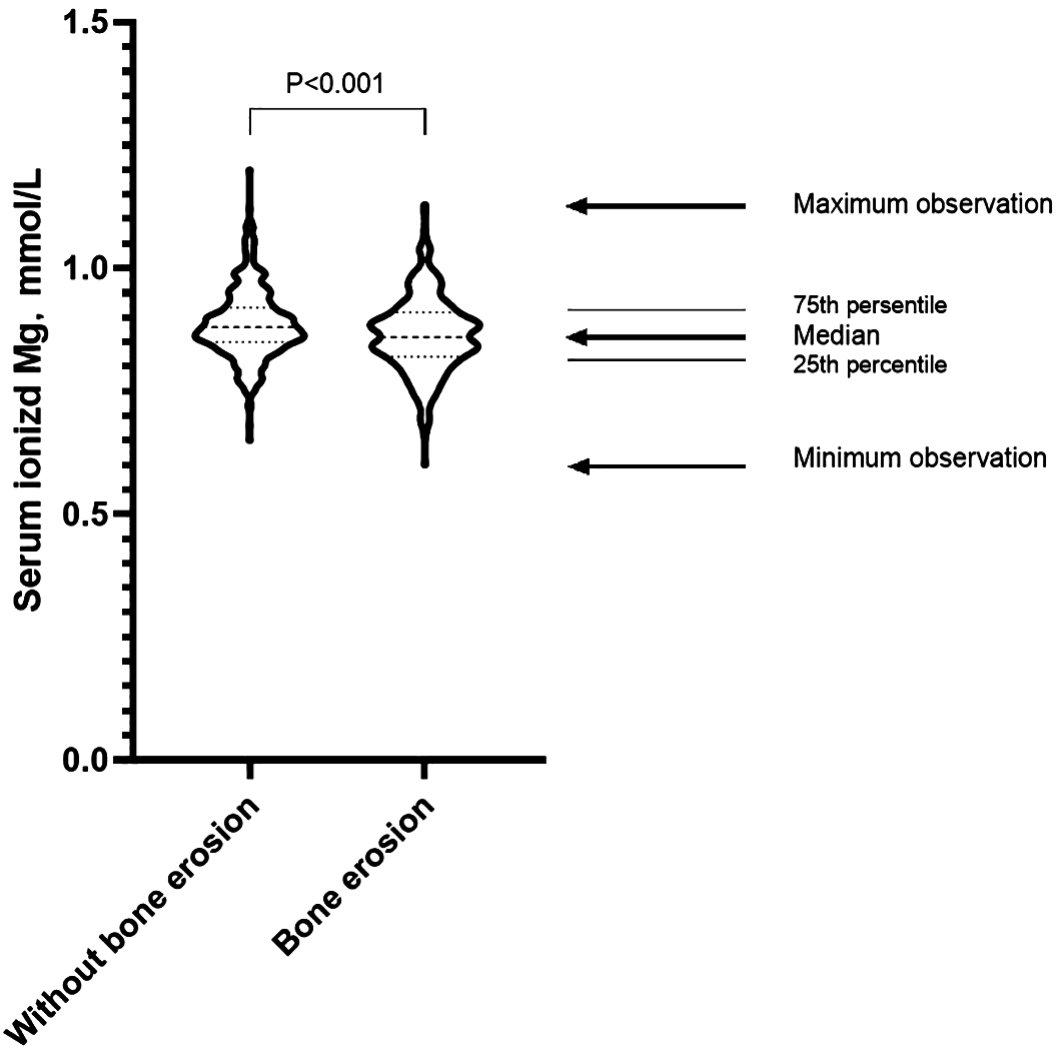
Distribution of serum ionized Mg levels in gout patients with and without bone erosion. Student’s t-test was employed to analyze the difference between the two groups. The serum ionized Mg level was significantly lower in the bone erosion group than the without bone erosion group (p<0.001).

The bone erosion group presented older age, longer gout duration, greater mean number of involved joints, significantly higher serum ionized Ca levels, and elevated ALP, PINP, OCN, and β-CTX levels. The bone erosion group also showed lower height and weight than the without bone erosion group. There was no statistically significant difference in the prevalence of osteoporosis between patients with and without bone erosion. (**Table 1**). Additionally, the serum ionized Mg level was significantly lower in the bone erosion group than the without bone erosion group (p<0.001, **Figure 3**).

### 3.2 The quartile stratification of ionized Mg in gouty arthritis patient

**Figure 4:**
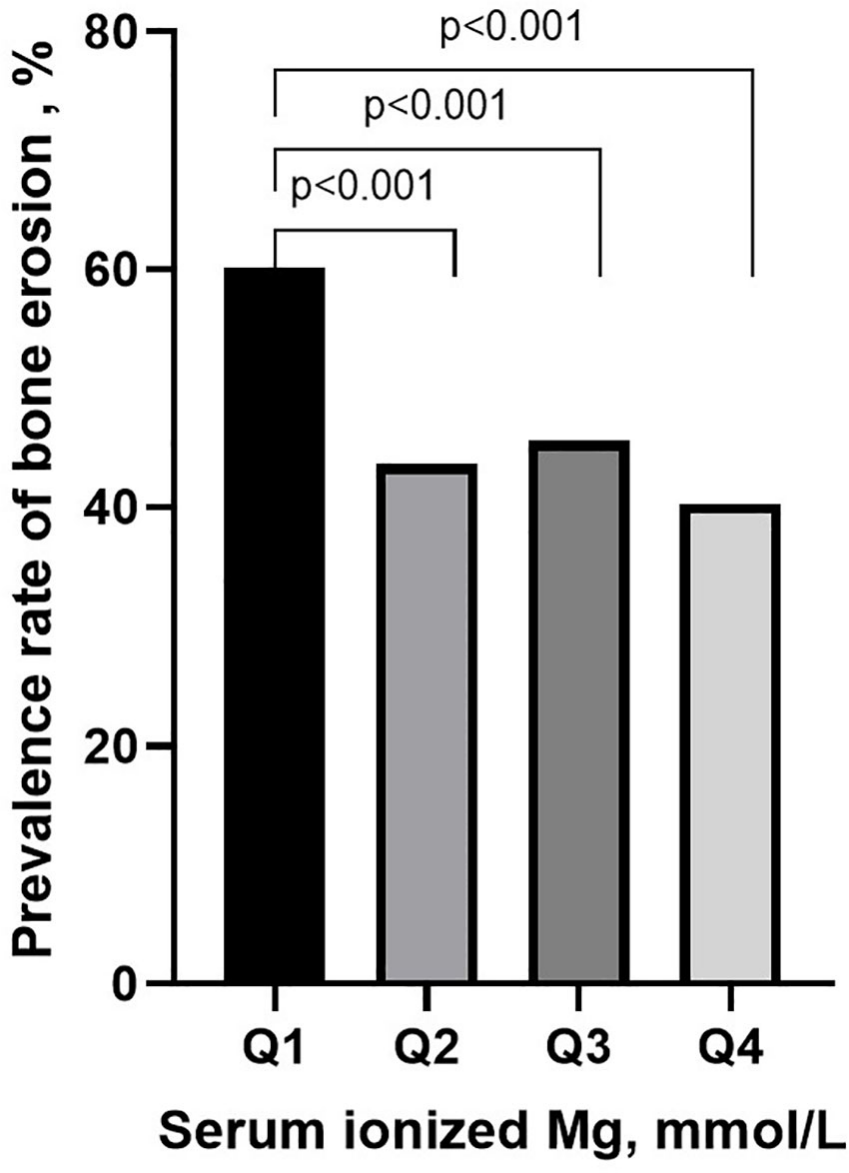
The prevalence of bone erosion in different groups of serum ionized magnesium levels. Group by quartile of serum ionized Mg: Q1: serum ionized Mg ≤0.83 mmol/L; Q2: 0.83<serum ionized Mg <0.88 mmol/L; Q3: 0.88≤serum ionized Mg <0.92 mmol/L; Q4: serum ionized Mg ≥0.92 mmol/L.

The clinical characteristics were analyzed in quartile stratification of ionized Mg in gout patients (**Table 2**). Compared with group Q4 (the patients with the highest ionized Mg level), group Q1 (the patients with the lowest ionized Mg level) exhibited a greater mean number of involved joints (p<0.01), and higher HbA1c (p<0.001) and FPG (p<0.05). The median of FPG in group Q2 was observed to be higher than that in group Q1. However, the difference between these two groups was not statistically significant. Both FPG levels in Q1 and Q2 were significantly higher than those in group Q4. Moreover, the prevalence rate of bone erosion in group Q1 was notably higher than in Q2, Q3, and Q4 (60.2% vs. 43.6%, 45.6%, 40.3%, p<0.001, **Figure 4**).

**Table 2:** Clinical characteristics of the study population stratified by serum ionized Mg quartiles in gout patients.

Characteristics	Q1 (≤0.83 mmol/L) N=201	Q2 (0.83-0.88 mmol/L) N=195	Q3 (0.88-0.92 mmol/L) N=182	Q4(≥0.92 mmol/L) N=191	P-value
General clinical data					
Age, y	51.8 ± 14.5	50.4 ± 15.7	52.6 ± 16.0	50.2 ± 16.1	0.373
Gender, male, %	188(93.5)	188(96.4)	175(96.2)	183(95.8)	0.497
Gout duration, y	8(3,15)	8(4,12)	7(3,15)	7(3,15)	0.401
Height, cm	173.8 ± 6.5	175.0 ± 6.47	173.8 ± 6.53	173.5 ± 7.2	0.141
Weight, kg	86.1 ± 18.5	88.6 ± 17.1	85.1 ± 15.3	84.7 ± 16.0	0.104
BMI, kg/m^2^	28.3 ± 5.1	28.8 ± 4.6	28.1 ± 4.2	28.0 ± 4.2	0.299
Waist, cm	102.6 ± 14.2	102.7 ± 13.5	99.8 ± 10.6	100.1 ± 11.4	0.056
Hip, cm	104.6 ± 9.9	105.8 ± 12.0	103.3 ± 7.6	103.3 ± 9.2	0.206
SBP, mmHg	141 ± 18	140 ± 18	139 ± 18	138 ± 15	0.268
DBP, mmHg	86 ± 12	87 ± 14	85 ± 12	85 ± 11	0.692
Radiographic features					
Bone erosion, %	121(60.2)	85(43.6)	83(45.6)	77(40.3)	<0.001*
Number of involved joints	3.1 ± 3.6	2.3 ± 2.4	2.3 ± 2.4	2.5 ± 2.7	0.005*
Joint effusion, %	107(53.2)	115(59.0)	103(56.6)	109(57.1)	0.710
Synovial thickening, %	103(73.6) a	115(85.2) b	105(81.4) a, b	98(73.1) a	0.038^
Tophi in soft tissues, %	43(21.4) a	33(16.9) a	16(8.8) b	31(16.2) a	0.009^
Biochemical profile					
HbA1c, %	5.9(5.5,6.7)	5.8(5.4,6.3)	5.7(5.4,6.1)	5.6(5.3,6.0)	<0.001*
FPG, mmol/L	5.18(4.59,6.26)	5.27(4.59,5.95)	4.91(4.50,5.55)	4.93(4.41,5.60)	0.002&
C peptide, ng/mL	3.72 ± 1.84	3.71 ± 2.08	3.62 ± 1.69	3.79 ± 1.57	0.859
Fasting insulin, uIU/mL	13.00(8.95,18.81)	13.50(9.11,17.45)	13.87(8.13,18.12)	13.90(9.70,18.88)	0.754
FFA, mmol/L	0.43 ± 0.17	0.41 ± 0.16	0.39 ± 0.17	0.41 ± 0.16	0.222
SUA, umol/L	471 ± 123	453 ± 124	461 ± 119	452 ± 111	0.350
SCr, umol/L	76(62,91)	73(63,88)	74(63,87)	75(63,88)	0.621
Ca, mmol/L	2.18 ± 0.18	2.15 ± 0.19	2.13 ± 0.19	2.15 ± 0.16	0.093
P, mmol/L	1.20 ± 0.20	1.19 ± 0.16	1.21 ± 0.19	1.25 ± 0.19	0.014^

Analysis of variance (ANOVA) test, Kruskal-Wallis H test, or Chi-square test.

*p<0.05.

^ The levels of serum P and the prevalences of synovial thickening and tophi in soft tissues varied between groups, but there was no significant difference between Q1 and Q4.

& The levels of FPG varied between groups. Both FPG levels in Q1 and Q2 were significantly higher than those in group Q4. The median of FPG in group Q2 was observed to be higher than that in group Q1. However, the difference between these two groups was not statistically significant.

Normally distributed variables are expressed as mean ± standard deviation. Non-normal variables are expressed as median (IQR). Categorical variables are expressed as percentages (%).

BMI, body mass index; SBP, systolic blood pressure; DBP, diastolic blood pressure; HbA1c, glycated hemoglobin; FPG, fasting plasma glucose; FFA, free fatty acid; SUA, serum uric acid; SCr, serum creatinine; Ca, serum ionized calcium; P, serum phosphorus; IQR, inter-quartile range.

### 3.3 The relationship of clinical features and serum Mg

We further analyzed the relationship between clinical characteristics and the serum ionized Mg levels (**Table 3**). Serum ionized Mg levels were negatively correlated with waist, hip, SBP, HbA1c, FPG, FFA, serum ionized Ca, and serum P (p<0.05). The negative correlations were particularly pronounced in HbA1c and FPG (p<0.001).

**Table 3:** Clinical characteristics correlated with serum ionized Mg (mmol/l).

Related variables	r	P-value
Age, y	-0.03	0.401
Gout duration, y	-0.048	0.181
Height, cm	-0.019	0.599
Weight, kg	-0.021	0.570
BMI, kg/m^2^	-0.031	0.388
Waist	-0.089	0.014*
Hip	-0.077	0.033*
SBP, mmHg	-0.079	0.028*
DBP, mmHg	-0.022	0.549
HbA1c, %	-0.189	< 0.001*
FPG, mmol/L	-0.128	< 0.001*
C peptide, ng/mL	0.024	0.512
Fasting insulin, uIU/mL	0.048	0.180
FFA, mmol/L	-0.071	0.049*
SUA, umol/L	-0.035	0.337
SCr, umol/L	-0.004	0.919
Ca, mmol/L	-0.072	0.047*
P, mmol/L	0.081	0.024*

Correlation was performed by Spearman correlation.

*p<0.05 indicates a statistical difference.

BMI, body mass index; SBP, systolic blood pressure; DBP, diastolic blood pressure; HbA1c, glycated hemoglobin; FPG, fasting plasma glucose; FFA, free fatty acid; SUA, serum uric acid; SCr, serum creatinine; Ca, serum ionized calcium; P, serum phosphorus.

### 3.4 The logistic regression analysis of risk factors for bone erosion

**Figure 5:**
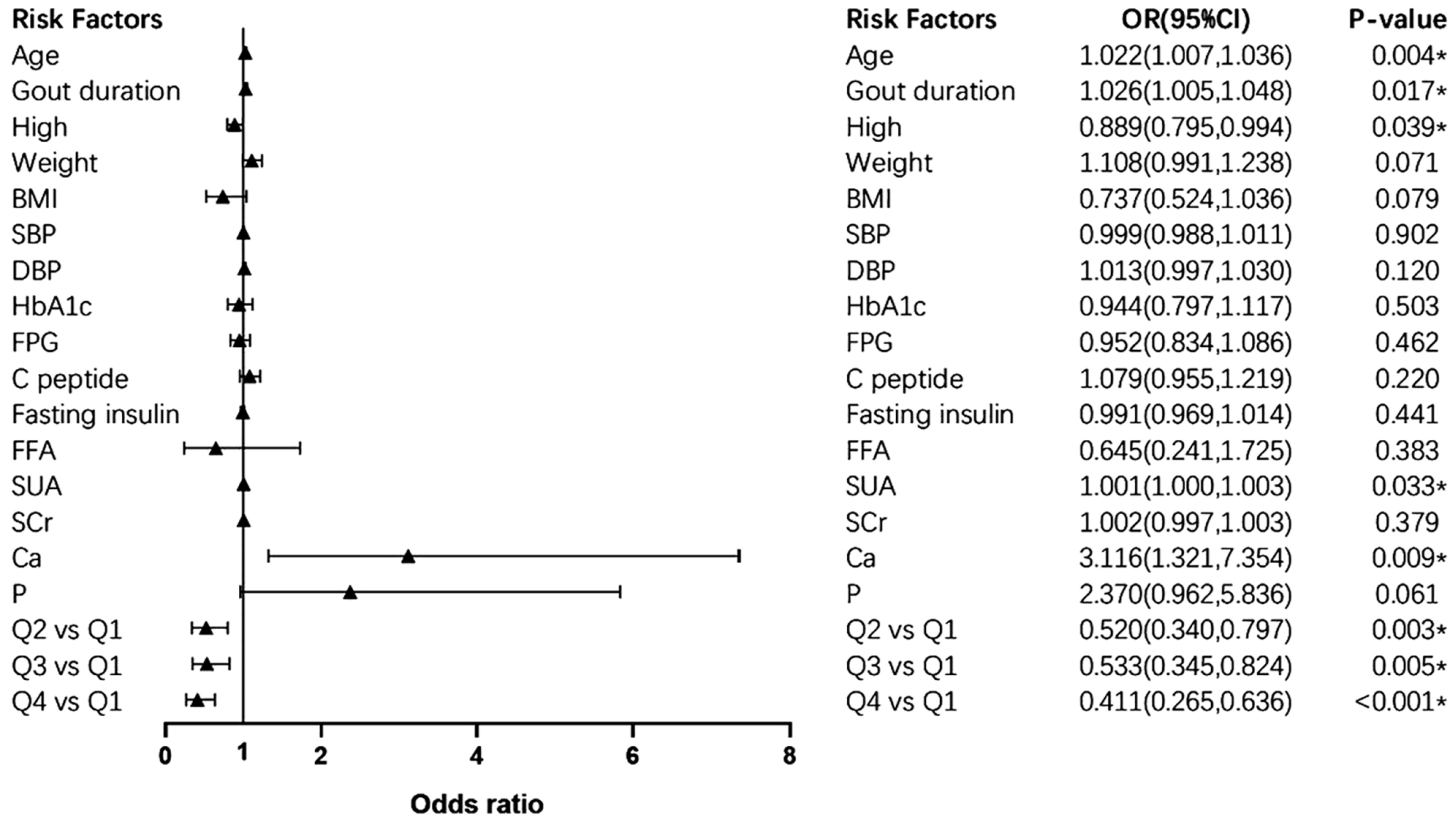
Forest plot of multivariate logistic regression. * presents statistically significant. OR (95%CI), odds ratio (95% confidence interval). SBP, systolic blood pressure; DBP, diastolic blood pressure; HbA1c, glycated hemoglobin; FPG, fasting plasma glucose; FFA, free fatty acid; SUA, serum uric acid; SCr, serum creatinine; Ca, serum ionized calcium; P, serum phosphorus.

The results of logistic regression were analyzed (**Table 4**). Compared with those in Q1, the odds ratios (95% confidence interval [CI]) for bone erosion were 0.511 (0.343, 0.762) in Q2 (p<0.01), 0.554 (0.369, 0.832) in Q3 (p<0.01), and 0.447 (0.298, 0.669) in Q4 (p<0.001). When adjusted for age, gout duration, height, weight, BMI, SBP, DBP, HbA1c, FPG, fasting insulin, FFA, SUA, SCr, serum ionized Ca and serum P, the significant association between serum ionized Mg levels and the risk of bone erosion persisted in Q2 (p<0.01), Q3 (p<0.01) and Q4 (P<0.001) with OR (95%CI) of 0.520 (0.340, 0.797), 0.533 (0.345, 0.824) and 0.411 (0.265, 0.636). The detailed multivariate logistic regression results are shown in **Figure 5**. Apart from serum ionized Mg, advanced age, long duration of gout, lower height, and high levels of SUA and serum ionized Ca are risk factors for bone erosion in gout patients.

**Table 4:** Unadjusted and multivariate-adjusted odds ratios (ORs) of the quartiles of serum ionized Mg levels for gout patients.

Quartiles	Unadjusted model		Adjusted model	
	OR (95%CI)	p-value	OR (95%CI)	p-value
**Q1**	–	–	–	–
**Q2**	0.511 (0.343, 0.762)	0.001*	0.520 (0.340, 0.797)	0.003*
**Q3**	0.554 (0.369, 0.832)	0.004*	0.533 (0.345, 0.824)	0.005*
**Q4**	0.447 (0.298, 0.669)	<0.001*	0.411 (0.265, 0.636)	<0.001*

Logistic regression analysis. Adjusted for age, gout duration, height, weight, BMI, SBP, DBP, HbA1c, FPG, Fasting insulin, FFA, SUA, SCr, Ca, and P.

*Statistically significant.

OR (95%CI), odds ratio (95% confidence interval).

## 4 Discussion

In this cross-sectional study, we examined the association between serum ionized Mg levels and bone erosion in 769 gout patients. The main finding was that patients with gouty arthritis who developed bone erosion had lower serum ionized Mg levels compared to those without bone erosion. The prevalence of bone erosion was higher in gout patients with the lowest serum ionized Mg levels compared to patients with relatively higher serum ionized Mg levels. Furthermore, multivariate logistic regression analysis showed that ionized Mg may be an independent protective factor for bone erosion in gouty arthritis. Assessing serum ionized Mg levels is of great significance in the management of patients with gout.

Previous clinical studies have investigated the relationship between Mg and bone health. Epidemiological studies have shown that dietary Mg deficiency may contribute to the development of osteoporosis (26). In a retrospective study targeting elderly populations, it was found that postmenopausal women with low bone density had lower serum Mg levels (27). Matias et al. showed that Mg intake is a significant and independent predictor of bone mineral density in young swimmers (28). Furthermore, Carpenter et al. conducted a prospective, year-long trial of magnesium supplements in children and adolescents, and their results showed that oral supplementation with magnesium oxide had a positive effect on the increase of hip bone mass in peripuberal Caucasian girls (29). It is also interesting to note that for patients with inflammatory bowel disease, mineral deficiencies, including magnesium deficiency due to poor intake or absorption, may contribute to the increased risk of osteoporosis (30). Mg is crucial for maintaining bone homeostasis, and its deficiency may be a factor that contributes to bone health issues in different populations.

The essence of bone erosion is the loss of bone mass (7). In this process, osteoclast-mediated bone resorption outweighs osteoblast-mediated bone formation (7). Indeed, the relationship between Mg and bone loss has been demonstrated in many basic studies. Here, we summarize several mechanisms that may explain our results.

1. Mg ions promote osteoblast proliferation, differentiation, and activity.

In *in vivo* experiments, it was found that Mg deficiency resulted in reduced number and activity of osteoblasts (31–33). These results were consistent with *in vitro* studies showing that high concentrations of Mg extracts advanced osteoblasts proliferation and differentiation (20). In addition, He et al. conducted a study that the treatment of osteoblast with Mg ions led to a significant rise in alkaline phosphatase and osteocalcin levels (34). These markers are widely recognized for indicating osteoblast function. Furthermore, Mg ions were observed to enhance gap junction intercellular communication (GJIC) between osteoblasts, stimulate osteoblast cell viability, and facilitate bone formation (34). Notably, certain molecular mechanisms associated with the aforementioned findings have also been reported. Leidi et al. showed that low Mg increased the release of nitric oxide (NO) by upregulating inducible nitric oxide synthase (iNOS), which inhibited the proliferation of osteoblast-like SaOS-2 cells and subsequently led to their decreased activity (35). Wang et al.’s research further demonstrated that Mg ions could activate the MAPK/ERK signaling pathway to promote the proliferation and differentiation of osteoblasts, thereby exerting their osteogenic potential (36).

2. Mg ions inhibit osteoclast formation, activity, and bone resorption.

Mg deficiency may enhance osteoclastogenesis, indicating a potential role for Mg in regulating bone remodeling (37). Wu et al.’s series of studies further demonstrated that low Mg concentration extracts could activate osteoclast activity, while high Mg concentration extracts could suppress it (20, 38). In addition, the mechanism underlying Mg’s anti-osteoclast activity was elucidated. The researchers demonstrated that Mg effectively inhibited the activation of nuclear factor-κB (NF-κB), a crucial transcription factor regulating osteoclastogenesis and bone resorption (39). This inhibition occurred through the retardation of inhibitor-κB degradation and subsequent NF-κB nuclear translocation (39). Moreover, Mg was observed to reduce the expression of NFATc1, which played a central role in osteoclast differentiation, maturation, and function, at both the protein and mRNA levels (39). These findings suggested that an imbalance in Mg ion homeostasis may result in abnormalities in bone absorption, thereby affecting the stability and integrity of the skeleton.

3. Mg indirectly affects bone homeostasis by influencing the actions of PTH.

It is well known that parathyroid hormone (PTH) plays a vital role in bone metabolism. High levels of PTH can promote bone catabolism, leading to bone loss and increased bone fragility (40). Rodríguez-Ortiz et al. evaluated the role of Mg in regulating parathyroid gland function using rat parathyroid glands *in vitro (* 41). These researchers observed that Mg could reduce the secretion of PTH when the parathyroid gland was exposed to moderately low concentrations of calcium (41). Furthermore, Mg could inhibit PTH secretion by inducing upregulation of parathyroid receptors, including the calcium-sensing receptor (CaSR), vitamin D receptor (VDR), and fibroblast growth factor receptor/Klotho (FGFR/Klotho) (41). In addition, the signal transduction of PTH involves the activation of adenylyl cyclase, a process that requires Mg as an essential cofactor (17). Thus, Mg not only reduces the secretion of PTH but also impairs the peripheral tissue response to PTH, which is beneficial for maintaining bone stability.

4. Mg affects bone homeostasis by participating in the regulation of inflammatory response.

The role of inflammation in promoting bone erosion and destruction has been widely recognized. In Mg-deficient rats, researchers observed an increase in the release of inflammatory cytokines, such as TNFα, IL-1, and IL-6 (42). These cytokines can amplify osteoclast function while inhibiting osteoblast function (17). Additionally, Mg deficiency can activate neuroendocrine pathways and trigger systemic stress responses by increasing the release of neuromediators like substance P (42). Notably, substance P released from bone nerve endings can also stimulate osteoclast activity, leading to bone loss (32). In brief, inflammation is an important link in the impact of Mg on bone homeostasis.

Combined with our results, a low serum ionized Mg level may be an important clinical feature in patients with gout who develop bone erosion. In the interquartile analysis, the lowest quartile serum ionized Mg (≤0.83mmol/L) showed a significant increase in the risk of bone erosion. These observational findings may support the hypothesis that Mg deficiency increases the risk of bone erosion in patients with gout. Multivariate logistic regression analysis further confirms that Mg is an independent protective factor for the occurrence of bone erosion in patients with gout. It suggests that Mg supplementation may be a novel approach to prevent bone erosion and maintain bone health in patients with gout.

Furthermore, we analyzed the relationships between serum ionized Mg with the biochemical parameters. It is important to highlight that serum ionized Mg shows highly significant negative correlations with HbA1c and FPG. Similar findings have been reported in people with type 2 diabetes or pre-diabetes (43, 44). Recently, Zhao et al. confirmed that the co-supplementation of chromium (Cr) and Mg could improve glycemic levels (45). However, this relationship between serum ionized Mg and blood glucose has not been reported in gout patients. The elevated risk of diabetes among gout patients has been well-established (46). Given the promising role of Mg in glycemic control, delving into the impact of serum Mg levels on blood glucose in gout populations would be valuable. Mg supplementation may provide more benefits to gout patients.

Overall, the maintenance effect of Mg on bone health has been widely recognized. Mg plays a role in reducing bone loss. Bone erosion, as an important early manifestation of bone loss, has significant implications for predicting and preventing muscle and bone dysfunction in patients with gout. At present, there is a lack of scholarly investigation examining the correlation between the Mg levels of individuals with gout and the occurrence of bone erosion. The present study is the first to fill this gap and offers novel insights for the early clinical management of patients with gout.

There is, of course, one caveat regarding our study. A high level of serum ionized Mg levels may contribute to bone protection, but it is not the higher the better. There is a lack of clinical research examining the impact of elevated serum Mg levels on bone health. However, *in vitro* studies have shown that excess Mg could reprogram the activity of VD3 in bone remodeling, leading to increased osteoclast differentiation and reduced osteoblast production (47). Therefore, our results support the notion that higher serum magnesium levels within the normal range may have protective effects on the skeleton and may reduce the risk of bone erosion in patients with gout. If Mg supplementation becomes a treatment option for preventing bone erosion in patients with gout, it is crucial to monitor serum Mg levels to avoid potential bone damage caused by excessive Mg levels.

Besides, there are some limitations in this study. Firstly, this is a cross-sectional study, which determines association, not causality. Prospective interventional studies are still needed to assess the effect of Mg on bone erosion. Secondly, serum ionized Mg levels are closely associated with dietary habits, but further analysis of dietary patterns cannot be conducted due to a lack of dietary data. Finally, our study population was >95% male. Whether these findings can be generalized to women is uncertain.

In summary, this study demonstrates a significant correlation between serum ionized Mg levels and the risk of bone erosion in patients with gout. The group of patients with bone erosion had longer durations of gout, more involved joints, and higher levels of bone metabolism markers such as ALP, PINP, β-CTX, and OCN. Patients with the lowest levels of serum ionized Mg had a greater number of involved joints and a significantly higher prevalence of bone erosion. Notably, higher levels of serum ionized Mg may independently protect against bone erosion in these patients. These results indicate that Mg supplementation may be a promising approach to prevent or slow down the development of bone erosion in these patients. However, further research is needed to confirm the effectiveness and safety of Mg supplements in treating gout.

## 5 Conclusion

The incidence of bone erosion is higher in gout arthritis patients with low serum ionized Mg levels. High serum ionized Mg levels may be an independent protective factor for bone erosion in gout arthritis.

## Data Availability

The raw data supporting the conclusions of this article will be made available by the authors, without undue reservation.
